# The Effects of Fat Content on the Shelf-Life of Vacuum-Packed Red Meat

**DOI:** 10.3390/foods13223669

**Published:** 2024-11-18

**Authors:** Elerin Toomik, Laura Rood, Ian Hunt, David S. Nichols, John P. Bowman, Chawalit Kocharunchitt

**Affiliations:** 1 Centre for Food Safety and Innovation, Tasmanian Institute of Agriculture, University of Tasmania, Private Bag 54, Hobart, TAS 7001, Australia; 2 Biomathematics and Statistics Scotland, James Clerk Maxwell Building, The King’s Buildings, University of Edinburgh, Edinburgh EH9 3FD, UK; 3 Central Science Laboratory, University of Tasmania, Private Bag 74, Hobart, TAS 7001, Australia

**Keywords:** pH, spoilage, beef, lamb, sensory qualities, microbial growth

## Abstract

When stored at chill temperatures, vacuum-packed (VP) lamb has a much shorter shelf-life than VP beef, primarily due to its higher pH, which could be linked to the higher fat content. The higher pH would create more favourable conditions for the growth of spoilage bacteria, resulting in a shorter shelf-life of meat. To determine the effects of fat on meat shelf-life as it relates to pH, a series of shelf-life trials at 2 °C were conducted using VP beef and lamb mince with varying fat contents (i.e., control with ~5%, 20%, and 50%) as a model system to red meat primal cuts. The results showed that higher fat content reduced the shelf-life of VP beef mince by 24% and lamb mince by 12.5%. This reduction was accompanied by significantly (*p* < 0.05) decreased glucose and lactic acid levels. Throughout storage, a higher fat content in beef and lamb mince generally resulted in a higher pH by 0.1 (*p* < 0.05) compared to the respective controls. Higher fat content mince also had faster lactic acid bacteria growth rates (by up to 0.13 Log10 CFU/g/day) and higher maximum populations of presumptive enteric bacteria up to 1.3 Log_10_ CFU/g (*p* < 0.05). These results suggest that fat content can negatively influence the shelf-life of VP red meat through lowering glucose and lactic acid levels, raising the pH, and increasing LAB growth rate and maximum population levels of presumptive enteric bacteria.

## 1. Introduction

The shelf-life of meat is determined by the growth and metabolism of the microbial community present and the subsequent production of spoilage compounds [1]. The growth of bacteria is affected by the intrinsic properties of meat (e.g., pH, glucose content, and fat tissue content), along with storage conditions [2] of which temperature is the most influential [1,3]. Under chill storage temperatures (−1 to 0 °C), the shelf-life of Australian vacuum packed (VP) lamb and beef primals is typically 13 and 28 weeks, respectively [3,4,5]. The shelf-life difference remains consistently evident across storage temperatures of 0–8 °C [6,7], which is thought to be driven by differences in the intrinsic pH of meat [5,7]. Specifically, lamb has a pH range of 5.6 to 6.8 depending on the cut, while the pH of beef is typically lower, falling between 5.5 and 5.8 [3,4,5,7,8]. The higher pH of lamb could be due to its higher fat content compared to beef (3.2–6.7% versus 1.7–5.2% wet wt) [9]. Indeed, it has been reported that fat has a higher pH (pH 6.5) compared to lean tissue of red meat [10,11]. Due to the distinct function and high lipid content of fat tissue, postmortem metabolic processes likely differ from those in lean meat [12] and do not lead to a reduction in pH levels as seen on lean meat. Meat with high pH (i.e., >5.8) can have reduced shelf-life due to the increased metabolic activity of bacteria [13] and elevated growth of high potential spoilage organisms (e.g., *Brochothrix thermosphacta, Serratia* spp., *Yersinia* spp., *Clostridium* spp., etc.) [7,14,15,16]. To support this further, there is anecdotal evidence from industry reporting that VP beef cuts with high intramuscular fat content (e.g., Wagyu and its crossbreeds with up to 50% intramuscular fat content) have a shorter shelf-life compared to leaner cuts.

To date, previous research has focused on the effects of fat in aerobically stored foods and food-based matrices, such as cheese, pâtés, fish emulsions, 3D matrices, etc. [17,18]. It is generally found that aerobic storage of high fat content (>20%) food products have shown a decrease in shelf-life due to chemical fat oxidation processes that cause rancidity [19,20]. However, spoilage of high fat content food under anaerobic conditions (e.g., VP red meat) is not due to chemical reactions but rather the activity of the microbial community that produce off-odours by consuming nutrients on meat (e.g., carbohydrates and proteins) [1,21,22]. There is currently limited research that has investigated the effects of fat on both the pH and microbial spoilage of VP red meat. Existing literature also shows inconsistent findings regarding fat content elevating VP red meat pH as the fat content increases [23,24]. These knowledge gaps highlight the need for more research to evaluate the effects of varying fat content on the shelf-life of VP red meat as it relates to meat pH and microbial growth. This will provide industry with scientific information about the shelf-life of different VP products allowing them to assure premium quality and reduce wastage.

The aim of this study was to investigate the effects of fat content on the shelf-life of VP beef and lamb at a low storage temperature. Minced meat was used as a model system for red meat primals. It is important to note that minced meat has an increased surface area compared to primal cuts and can promote the growth of bacteria and reduce subsequent shelf-life. However, the use of mince enables easier control of localised variances in pH and fat content across a meat cut. This provides a better understanding of the effects of fat on the shelf-life of meat. Throughout storage, beef and lamb mince samples with varying fat levels were periodically assessed for sensorial qualities, chemical properties (e.g., glucose and lactic acid levels), pH, and bacterial growth. This was performed to confirm the detrimental effects of fat content on the shelf-life of red meat.

## 2. Materials and Methods

### 2.1. Beef and Lamb Mince Collection

In this study, two shelf-life trials were conducted independently to evaluate the shelf-life of either VP beef or lamb mince with different fat content (20% and 50% *w*/*w*). The 50% fat content was chosen to model the effect of the highest expected intramuscular fat content found in red meat, e.g., in Wagyu [25], while 20% fat content was chosen as a representation of typical Australian lamb cuts [26].

A single batch of fresh beef and lamb mince (8.5 kg, 95% chemically lean per producer’s specifications) and their respective fat (3.0 kg), were sourced from a local producer (Tasmania, Australia). In both instances, mince was produced from a combination of lean trims from carcasses as per standard processing procedure. Fat was sourced as minced fat from subcutaneous fat tissue trimmings. All lean and fat trims were from a single herd of animals slaughtered 2 days prior. Immediately after processing, the mince was transported under chill temperatures to the University of Tasmania’s Microbiology Research Laboratory for commencement of trials (i.e., within an hour of mincing).

### 2.2. Preparation of Mince with Different Fat Content

**Figure 1:**
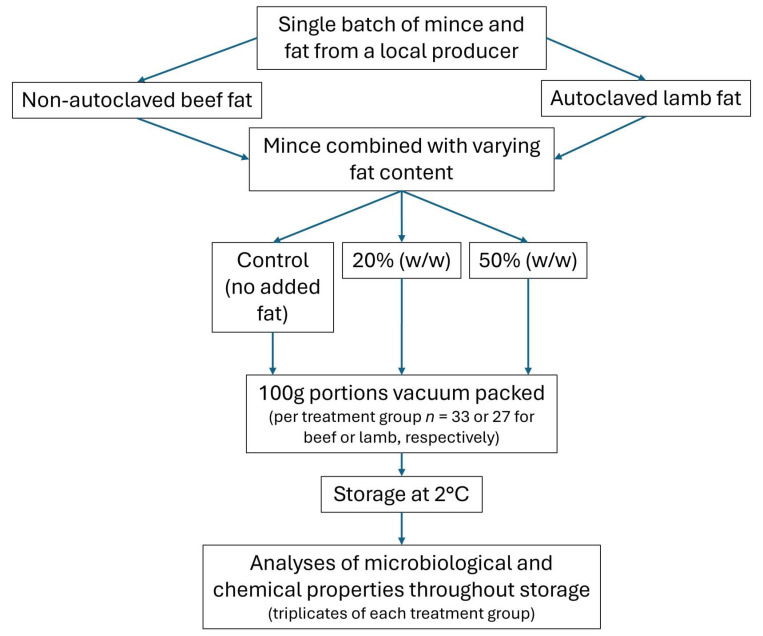
Experimental design flowchart of sample processing steps in the laboratory for the independent beef and lamb mince trials.

Under aseptic conditions, appropriate proportions of lean mince and fat were weighed and combined to produce mince with the final fat content of 20% and 50% (*w*/*w*) (total of 4.29 kg of each) with the processing steps represented in Figure 1 for both independent beef and lamb trials. Beef and lamb mince with no added fat were used as controls. Additional steps were included for the lamb trial, in which the fat was autoclaved at 121 °C for 25 min (Tomy SX-700E, Tomy Kogyo Co Ltd., Tokyo, Japan) and cooled in an ice bath for 30 min before addition to the minced meat. This was to minimise the variations in the initial bacterial numbers of each treatment group due to different amounts of fat added, as differences were observed in the first trial using beef mince (see Section 3.3).

To ensure adequate distribution of fat throughout the mince, lean mince and minced fat were homogenised by hand with sterile gloves for 5 min for all treatment groups. Portions of a 100 g of each sample (beef *n* = 33; lamb *n* = 27 per treatment group) were aseptically placed into Cryovac TBG4620 (CRYOVAC®, Sydney, Australia) barrier vacuum packs (O_2_ transmission rate of 20 cc/m^2^/24 h at 23 °C, 0% relative humidity) and sealed with a tabletop Tecnovac T60 vacuum machine (Tecnovac S.R.L., Grassobbio, Italy) with vacuum set to 99.9% and sealing set to 2 s. For both beef and lamb trials, samples were stored at 2 °C, and throughout the shelf-life three replicates for each treatment group were periodically withdrawn for analyses (see Section 2.3). Sampling times for beef were on storage days 0, 2, 5, 7, 11, 15, 22, 25, 28, 33, 34, and 39, and sampling times for lamb were on storage days 0, 2, 5, 8, 12, 14, 16, 20, and 26. Minced beef and lamb fat samples (*n* = 12 per trial, 40 g each) were vacuum-packed and stored as described above and were sampled at the beginning and end of the shelf-life trials.

### 2.3. Sensory Evaluation, pH, and Microbiological Analyses

Packs were randomly chosen for assessments and were visually examined to exclude packs that had lost their vacuum. The shelf-life assessment was conducted in accordance with the standardised protocols used by the Australian red meat industry [4,6,7,27] with some minor modifications, as described below. Sensory qualities of each sample were assessed by a semi-trained panel of 3 to 6 people over the course of storage. Prior to the trials, a practice training session was held for the assessment of samples at different shelf-life stages. Panellist were from University of Tasmania (staff and students) with diverse backgrounds to represent consumers. 

Persistent odour was assessed 10 min after aseptically opening the pack as previously described [6,7,28]. Briefly, odour was assessed on a 9-point categorical hedonic scale (0 = extreme off-odour, 2 = strong off odour, 4 = moderate off-odour, 6 =very slight sour odour, 8 = no odour, normal meaty odour, “fresh”). Samples with a score of ≤4 were considered spoiled and commercially unacceptable [28]. Minced fat samples were not subjected to sensory analyses.

The pH of samples (mince with different fat content) was measured using a handheld pH meter (Model 206-pH2, Testo, Kilsyth, Australia) according to the manufacturer’s instructions. Four measurements were taken at random locations in the mince pack. Minced beef and lamb fat pH was measured only at the beginning of the trials.

To enumerate bacteria, 10 g of sample was transferred aseptically to a sterile bag (532 mL Whirl-Pak^®^, Nasco Sampling, Madison, WI, USA) and 90 mL of sterile 0.1% peptone water (Oxoid, L37, Thebarton, Australia) was added and homogenised for 1 min to create a rinsate. Serial dilutions were prepared from the rinsate in sterile 0.1% peptone water and spread plated. Total viable counts (TVC) as per standardised approach by the Australian red meat industry [5,6,7,27] were enumerated on tryptone soya agar (TSA; Oxoid, CM0131, Thebarton, Australia), while lactic acid bacteria (LAB) were enumerated on de Man–Rogosa–Sharpe agar (MRSA; Oxoid, CM0359, Thebarton, Australia). Presumed enteric bacteria (family *Enterobacteriaceae* and its closely related organisms), regardless of the lactose reaction, were enumerated on MacConkey agar (MAC; Oxoid, CM0115B Thebarton, Australia). TSA and MRSA plates were incubated for 5 d at 25 °C and MAC plates were incubated for 2 d at 37 °C. MRSA plates were incubated under anaerobic conditions using Anaerogen Compact pouches (AnaeroGen™ 3.5 L, Thermo Scientific, Thebarton, Australia). Microbial counts for minced fat were only enumerated at the beginning and end of the trials.

### 2.4. Determination of Mince Fat, Glucose, and Lactic Acid Content

A sub-sample of approximately 10 g of beef and lamb mince was transferred to sterile 15 mL plastic test tubes (Greiner Bio-One™ CELLSTAR™ Test Tubes, 188261, Kremsmünster, Austria). These subsamples were collected on storage day 0, 15, and 33 for beef mince treatments, and day 0, 8, and 20 for lamb. Beef and lamb fat samples were sub-sampled only at the beginning of the trials. All samples were snap frozen with liquid nitrogen for 5 min and stored at −80 °C. Upon completion of the shelf-life trials, these samples were used to determine fat, glucose, and lactic acid content. Prior to analyses, the samples were thawed for 15 min at room temperature.

#### 2.4.1. Mince Crude Fat Content

The total crude fat content (%) of the samples was measured using solvent extraction method with Ankom XT15 Extractor (Macedon, NY, USA) following the operator’s instructional procedure [29]. Briefly, approximately 1.5 g of sample was weighed into Ankom specific filter bag (XT4, Ankom Technology, Macedon, NY, USA), sealed, and dried for 3 h at 102 ± 1 °C. The crude fat of the dried sample was then extracted with petroleum spirit (BSPPL734.2.5, LabServ, Thermo Fisher Scientific, Scoresby, Australia) in the fat extractor for 60 min at 90 °C with a water level gauge set at 6.5. After extraction, the sample was dried for 15 min at 102 ± 1 °C to evaporate any residual solvent. The crude fat content was determined gravimetrically based on the fat lost from the sample in the drying and extraction processes.

#### 2.4.2. Mince Glucose and Lactic Acid Concentrations

To measure glucose and lactic acid content, 0.05 g of mince sample was added to cold 1:1 (*v*/*v*) acetonitrile (34851, Sigma-Aldrich, Bayswater, Australia) and methanol (34860, Sigma-Aldrich, Bayswater, Australia) mixture followed by spiking with 25 μg of ^3^H_2_ labelled L-lactic acid (L113503, Toronto Research Chemicals, North York, ON, Canada) and 18 μg ^13^C_6_ labelled D-glucose (CIL-CLM-1396-CTM, Cambridge Isotope Laboratories, Inc., Andover, MA, USA) to make a final volume of 1 mL. The mince was homogenised (DLAB D-160, Beijing, China) on high speed for approximately 1 min. After vortexing, the samples were ultrasonicated (Grant XUB Digital ultrasonic bath, VWR International, Pty Ltd., Tingalpa, Australia) in ice cold water for 30 min, followed by 20 h incubation at −20 °C to precipitate proteins. Samples were then centrifuged at 14,000× *g* for 30 min at 4 °C, the supernatant was collected and stored at −20 °C until analysis.

##### Ultra Performance Liquid Chromatography–Mass Spectrometry Analysis

Glucose and lactic acid concentrations in samples were determined in comparison to known concentrations of the stable isotope labelled standards (as described above). The concentrations of the standards were selected based on preliminary assessment of the linearity between concentrations and corresponding chromatographic peak areas. For these analyses, a Waters Acquity H-class ultra-performance liquid chromatography (UPLC) system (Waters Corporation, Milford, MA, USA) was utilised. The system was equipped with an Acquity BEH Amide VanGuard pre-column (5.0 × 2.1 mm, 1.7 μm) and an Aquity BEH Amide column (2.1 × 150 mm × 1.7 μm) (Waters Corporation, Milford, MA, USA). Chromatography was performed with a mobile phase consisting of 0.4% (*v*/*v*) ammonium hydroxide (Solvent A) and acetonitrile (Solvent B). Elution was completed using a solvent gradient series. For this, initially solvent B at 80% was run before switching to a gradient of 71.7% solvent B over 2 min, which was held for a subsequent 2 min. The system was returned to initial conditions after 4.5 min and re-equilibrated for 3 min. The flow rate was 0.35 mL/min and the column was held at 45 °C. Injection volume was 1 μL. The typical retention times observed for lactic acid and glucose were 1.2 and 4.3 min, respectively.

The UPLC was coupled to a Waters Xevo TQ triple quadrupole mass spectrometer (Waters Corporation, Milford, MA, USA). Analyses were undertaken using multiple reaction monitoring (MRM) in negative electrospray ionisation mode, with one MRM transition monitored for the analyte of interest and the stable isotope labelled standard, respectively. Electrospray ionisation was performed with a capillary voltage of 2.5 kV, and individual cone voltages (V) and collision energies (V) for each MRM transition as described in Table 1. The desolvation temperature was 450 °C, nebulising gas was nitrogen at 950 L/h, and cone gas was nitrogen at 100 L/h. MRM transition dwell times were 120 msec.

### 2.5. Statistical Analysis

The shelf-life of mince products was determined based on an average odour score reaching ≤4 at a given and subsequent timepoint, as previously described by Rood, Bowman, Ross, Corkrey, Pagnon, Kaur, and Kocharunchitt [28].

The statistical analyses were performed separately for both beef and lamb trials given that they were conducted as independent experiments. Specifically, the measurements of fat content, glucose, and lactic acid content were modelled using generalised linear regression models (GLS) using R (v.4.01). A different GLS model was estimated using each of these measurements as a y-variable (making six models in total). Each model had the following x-variables: the storage day of sampling (categorical), the treatment (categorical, according to the fat content) and an interaction term between these two variables. For each model, the variance of residual errors was estimated separately for each treatment. The GLS excluded the measurements for beef and lamb fat samples as they were only measured at the beginning of the storage. Other measurements excluded from the models were glucose content at storage days 15 and 33 for beef and day 20 for lamb as the levels fell below detection limit. Alongside these models, all pairwise treatment contrasts were estimated (from models that excluded the interaction terms). The *p*-values for the contrasts were adjusted for multiplicity using a Bonferroni adjustment. All model residuals were checked for approximate identical and independent normality using QQ-plots (per treatment group).

Using R and the ‘multilevel’ package, linear mixed models (LMM) were estimated for beef and lamb mince pH data. Separate LMMs were estimated for each storage day. Each model had the pH level as a y-variable, ‘replicate’ as a random effect, and the x-variable fixed-effects were from a categorical variable indicating the treatment level. Within each model the data came from three treatments with three replicates each; from each replicate, four different pH measurements were made (*n* = 36 for each LMM). The models were used to estimate the pairwise differences between each of the respective treatments. *p*-values from asymptotic Wald tests were calculated, where each null hypothesis is that the difference between a pair of treatments is truly zero. 95% confidence intervals were estimated for the pairwise differences between treatment means and a *p*-value of <0.05 was considered significant.

Separate growth curves for beef and lamb mince controls, 20%, and 50% were constructed by plotting the triplicates of count data (Log_10_ CFU/g) against time (i.e., storage day; *n* = 10) to establish exponential growth rates and lag times. At minimum, 3 timepoints were used in the exponential growth phase to fit a linear regression line with minimum acceptable R^2^ value of 0.95 as described by Kocharunchitt et al. [30]. The calculated slope of the regression line was the growth rate (Log_10_ CFU/g/day). Lag time (in days), where applicable, was estimated from the point where the regression line first passed through the growth curve [30]. The maximum population was taken as an average across the stationary phase count data.

The separate microbial indicators’ (i.e., TVC, LAB, presumptive enteric bacteria) initial and maximum counts for all the treatment groups were analysed with one-way ANOVA using R with treatment as a factor and count data as the variable. Tukey’s HSD (Honest Significant Difference) post hoc test was used to determine significant differences (*p* < 0.05) between groups of comparisons.

All the boxplots were plotted using R with the ‘ggplot2’ package [31], where the central box represents the interquartile range (IQR) with the median marked by the horizontal line in the box. Whiskers extend to 1.5 times the IQR and beyond the whiskers are separate outlier points.

## 3. Results and Discussion

Beef and lamb trials were carried out on two separate occasions. The same procedures were used to determine their shelf-lives with an exception that fat was autoclaved before mixing with mince in the lamb trial. Accordingly, direct comparisons between beef and lamb regarding fat content, shelf-life, pH, and microbial growth were not made in this study.

Each treatment group of VP beef and lamb mince stored at 2 °C had fat content (*w*/*w*) as expected, the fat content did not significantly change over time for beef (F_2,2_ = 0.44, *p* = 0.65) or lamb (F_2,2_ = 0.62, *p* = 0.55; ANOVA summaries also provided in Supplementary Table S1). Accordingly, the fat content was averaged for each treatment group and presented in Table 2.

### 3.1. Shelf-Life of VP Red Mince

The shelf-life of the mince products at 2 °C was determined in accordance with previous studies that have demonstrated odour as the main microbial spoilage parameter of raw VP red meat at cool temperatures (≤4 °C) [4,6,7,8,27,32,33,34]. The storage temperature of 2 °C shortened the expected time of spoilage while minimising significant changes to the microbial community composition compared to the ideal storage at 0 °C [7,28].

In all cases, the VP mince with added fat had a shorter shelf-life compared to the respective controls based on the odour scores (see Supplementary Figure S1 for specific odour scores). However, there was no difference in the shelf-lives between the 20% and 50% fat treatments for each meat type. Specifically, mince containing 20% and 50% fat content both had a shorter shelf-life by 24% (33 vs. 25 days) for beef and 12.5% (16 vs. 14 days) for lamb compared to the control. This agrees well with the study of Blixt and Borch [35] showing that higher fat content in VP pork (~16%) and beef (~12%) mince led to faster spoilage (assessed by sensory panellist) compared to respective mince from leaner cuts (4% vs. 8%) during storage at 4 °C. It has also been shown that VP lamb mince with 20% fat content had increased production of spoilage compounds (e.g., acetaldehyde, 2-propenal, 2-butanone) compared to lean mince stored at 2 °C [24], likely resulting in shorter shelf-life. The basis of the apparent similarities in the shelf-lives of mince containing 20% and 50% fat across the two independent trials for beef and lamb is unknown. Further studies with smaller increments of fat added (e.g., 5%, 10% and 15%) would provide deeper insights into the level of fat content that significantly affects the rate of quality loss.

### 3.2. Intrinsic Properties of VP Mince

#### 3.2.1. Glucose and Lactic Acid Levels

**Figure 2:**
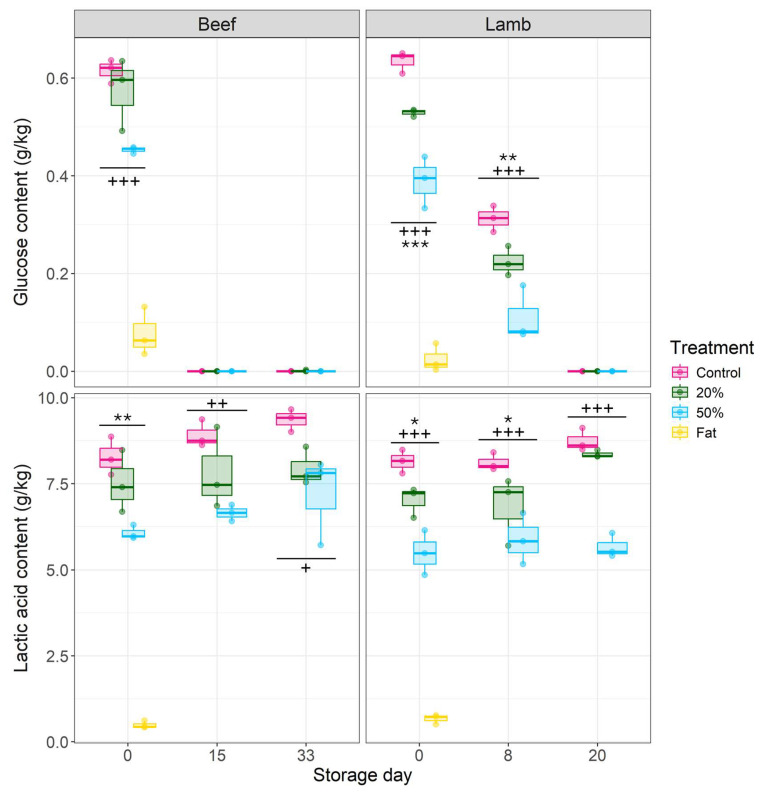
Boxplots of glucose (above) and lactic acid (below) content (g/kg wet wt) of VP beef (on left) and lamb (on right) mince with varying fat content (*w*/*w*) stored at 2 °C. Measurements (*n* = 3) were taken at storage day 0, 15, 33, and 0, 8, and 20 for beef and lamb, respectively. Fat samples (*n* = 3) were only measured at day 0. Statistical differences based on GLS pairwise contrasts are indicated by * *p* < 0.05, ** *p* < 0.01, *** *p* < 0.001 for 20% vs. control and + *p* < 0.05, ++ *p* < 0.01, +++ *p* < 0.001 for 50% vs. control at a given timepoint. The lower detection limit of glucose and lactic acid was 0.00045 and 0.00083 g/kg, respectively.

The initial beef and lamb control mince glucose levels were both approximately 0.6 g/kg (Figure 2). These levels were on the lower end of that typical to red meat, which can range from 0.17 to 1.5 g/kg depending on the breed and welfare of the animal before slaughter [27,36]. On the other hand, the initial lactic acid content fell within the typical range for red meat [37,38], which were 8.28 ± 0.56 g/kg and 8.15 ± 0.34 g/kg for beef and lamb control, respectively (Figure 2).

The initial glucose content significantly decreased (*p* < 0.001) as the fat content increased in both beef and lamb (Figure 2). Similarly, the lactic acid content was significantly lower (*p* < 0.01) in 50% beef and lamb mince compared to the respective control regardless of storage day (Figure 2). These results were likely due to the substantial addition of fat to mince, where both fat samples had around 10-fold lower glucose and lactic acid content compared to control mince (Figure 2). Fat tissue has low glucose and lactic acid levels due to its physiology and function [39]. Glucose is an important energy source for bacteria on meat and its utilisation typically does not lead to offensive off-odours [1,27]. Indeed, the importance of glucose levels on meat shelf-life have been previously shown on VP lamb stored at 4 °C, where addition of glucose solution (up to 0.4 g/kg) extended the shelf-life of the product by ≥76% (i.e., >13 days) [27]. This suggest that the initial lower glucose levels in high fat content beef and lamb mince could explain earlier spoilage of high fat content beef and lamb mince (Section 3.1). Specifically, the spoilage bacteria had limited glucose availability and began utilising proteins and amino acids, leading to earlier accumulation of offensive off-odours [1,40].

Depletion of glucose below the detection limit (<0.00045 g/kg) during storage (Figure 2) was expected as it is preferred energy source for bacteria to grow on meat [40]. Metabolism of glucose on meat also leads to accumulation of organic acids, especially lactic acid, due to the activity of various lactic acid bacteria over the course of anaerobic storage, resulting in pH decrease [41,42]. However, the lactic acid content did not significantly increase (*p* > 0.01) during storage, except for 20% lamb mince (day 8 vs. day 20, *p* = 0.008, 95%CI −2.67 to −0.36). Some of the changes in lactic acid content through time could have been missed due limited measurements or due to low glucose levels (Figure 2). The lack of lactic acid accumulation could also have been due to minimal and non-detectable production of lactic acid and/or production of other organic acids that were not measured. Various bacteria (such as lactic acid bacteria) can also utilise lactate for their growth during storage, depleting its levels at the same time [43,44].

#### 3.2.2. pH

**Figure 3:**
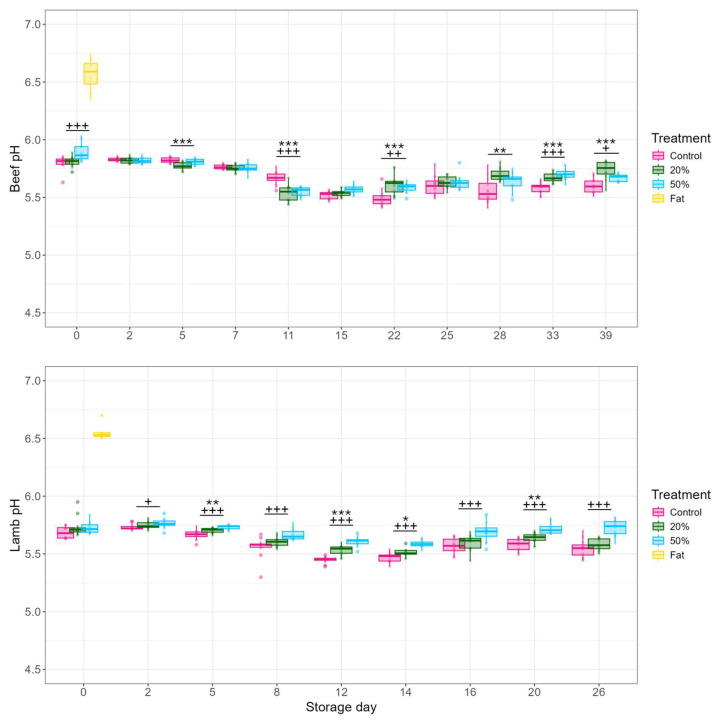
Boxplots of pH values of VP beef (**top**) and lamb (**bottom**) mince with varying fat content over the course of storage at 2 °C. Each storage day is represented by triplicates with four measurements (*n* = 12) for each treatment. Fat samples (*n* = 3) were only measured at day 0. Statistical differences based on LMM pairwise contrasts are indicated by * *p* < 0.05, ** *p* < 0.01, *** *p* < 0.001 for 20% vs. control and + *p* < 0.05, ++ *p* < 0.01, +++ *p* < 0.001 for 50% vs. control at a given timepoint.

The initial pH levels on beef and lamb mince depicted in Figure 3 agree with previous findings on red meat lean and fat tissues [7,10,34]. Despite the significant addition of fat (20% and 50% *w*/*w*), throughout storage the pH levels remained within the expected range (5.5–5.8) previously observed in red meat [1,7,23,42,44,45,46].

Both 20% beef and lamb mince initial pH levels were not significantly different from the respective controls, which agree with previous findings [23,24]. Addition of 50% fat to beef mince significantly (*p* < 0.001) raised the average initial pH by ~0.1 compared to the control, but not with lamb mince (Figure 3). During storage, significant effects of fat on beef mince pH were not observed again until day 15, after which both 20% and 50% beef mince were significantly (*p* < 0.05) higher from the control until the end of the storage (except day 25; Figure 3). For lamb, this trend was observed from storage day 5 (Figure 3). Similarly, Schuster, Franke, Silcock, Beauchamp, and Bremer [24] observed significantly higher pH for VP lamb mince containing 20% fat compared to that of lean mince (5.75 vs. 5.62) by 14 days of storage at 2 °C, which was accompanied by increased production of spoilage compounds. These results suggest that fat with a high pH of 6.5 (Figure 3) can contribute to an increase in meat pH. This in turn favours the growth of various spoilage bacteria, such as *Hafnia* sp., *Yersinia* sp., *B. thermosphacta, Clostridium* spp., etc., [3] and ultimately leads to earlier spoilage.

It is acknowledged that the changes in the pH for both beef and lamb between control and mince with added fat throughout storage were small (~0.1; see Supplementary Tables S3 and S4 for specific average differences at each timepoint), but they were significant (Figure 3). The relatively small changes in pH values could have stemmed from meat having high buffering capacity [47,48,49]. However, pH changes (of ≤1 pH unit) can have substantial systemic effects on microbial metabolism [13,50,51] and their growth rates [15,52], which can influence red meat shelf-life. The effects of fat and the subsequent rise in pH on microbial growth kinetics are discussed further in Section 3.3.

### 3.3. Bacterial Growth Kinetics

The initial populations on beef mince ranged between 4.50 and 5.00 Log_10_ CFU/g for TVC, and 3.15 and 3.46 Log_10_ CFU/g for presumptive enteric bacteria, with the latter showing a non-significant (*p* > 0.05) difference with varying fat content (Table 3). Similar trends were also observed for the initial LAB populations, which ranged between 3.69 and 4.67 Log_10_ CFU/g. However, there was a systematic increase in LAB numbers with increasing fat content (*p* < 0.05; Table 3). This is likely explained by the higher microbial load observed on beef fat samples that was added to the mince (Table 3).

Lamb mince initial bacterial populations did not increase with the addition of fat (Table 3) due to prior sterilisation of lamb fat. The initial LAB and presumptive enteric bacteria populations ranged from 3.76 to 4.02 and 3.07 to 3.38 Log_10_ CFU/g, respectively (Table 3). The initial TVC population on lamb mince was higher compared to the beef, ranging between 5.22 and 5.77 Log_10_ CFU/g (Table 3). As beef and lamb trials were conducted on separate occasions, the higher initial population load on lamb compared to beef (despite both being freshly produced and sourced) may originate from differences in handling and mincing steps by the producer at the given day. Furthermore, it could have also been a result of more labour-intensive slaughtering procedures required for lamb with smaller carcasses compared to beef processing [7,53,54].

Despite sterilisation (121 °C for 25 min) prior to commencement of the trial, lamb fat still had an initial bacterial load of 3.65–4.58 Log_10_ CFU/g across bacterial indicators (Table 3). This was not expected and could have been due to the intrinsic nature of fat tissue (3.0 kg) requiring longer sterilisation time of up to 40 min [55] compared to what was used in this study to achieve complete elimination of bacteria.

The higher initial bacterial populations on 20% and 50% beef mince compared to the control might have contributed to their shorter shelf-lives (Section 3.1). However, the differences in these initial populations between treatments were small (<1 Log_10_ CFU/g), and only 50% mince was significantly higher (Table 3). In the case of lamb mince with 20% and 50% fat content, which had similar initial bacterial loads, the shelf-life was still shorter than the control (Section 3.1).

Unexpectedly, beef and lamb control mince TVC had a lag phase of 3.65 days and 1.81 days, respectively, while the 20% and 50% treatments did not. The basis of this remains to be elucidated, as lag phase is not typically observed with VP red meat under chilled conditions [7,11].

In all cases, with the possible exception of 20% lamb mince, the growth rates of TVC decreased as fat content increased while the growth rates of LAB were faster with increased fat content (Table 3). This inconsistent trend between TVC and LAB was unexpected, given that LAB are the dominant part of the microbial community of VP red meat products [7,23,56]. LAB species dominate VP meats because they can grow rapidly under anaerobic conditions at ≤4 °C, produce acidic by-products reducing meat pH, and, therefore, outcompete other higher spoilage potential bacteria, such as *Brochothrix thermosphacta* and various enteric bacteria [44,57,58]. Indeed, the results also showed that LAB made up ~96% of the TVC by day 11 and 8 of storage (Supplementary Figure S2) when maximum population was reached on beef and lamb mince, respectively. This indicates the differences in growth rates could be instead due to the different incubation conditions between TVC and LAB media plates, where the latter was incubated anaerobically and was more suitable for LAB growth.

Nonetheless, the faster growth rate of LAB on mince with higher fat content could have potentially contributed to the shorter shelf-life compared to the control mince. Faster growth rates of LAB can lead to greater competition for preferentially used glucose [1], especially on high fat content mince with reduced glucose levels (Figure 2). This leads to the utilisation of proteins and/or amino acids as an alternative carbon source, increasing meat pH due to accumulation of alkaline by-products [3] and causing earlier production of off-odour compounds [1,59]. Indeed, certain LAB species have been shown to cause red meat spoilage [28] by producing spoilage related compounds, such as acetate, ethanol, butanol, 2,3-butanediol, acetoin, and diacetyl with sour off-odours [22,56,59,60,61]. On high fat content mince, some LAB could have also broken down triglycerides from fat to produce energy [39,62], leading to accumulation of free fatty acids, aldehydes, ketones, and short chain fatty acids that can negatively affect the odour profile of meat [63].

Generally, there were no differences in the maximum populations of either TVC or LAB between respective beef and lamb mince treatments (Table 3). However, the maximum population of presumptive enteric bacterial counts increased (*p* < 0.05) with fat content for both beef and lamb, with the fat samples having the highest maximum populations (Table 3). These results indicate that higher fat content can support elevated populations of enteric bacteria and may have contributed to the shorter shelf-life of high fat content beef and lamb mince. Previous research has found that enteric bacteria (e.g., *Yersinia* spp., *Hafnia* spp. *Serratia* spp., *Providencia* spp.) can reach higher maximum population densities on VP beef adipose tissue compared to lean meat when stored at 5 °C, most likely due to the former having higher pH [46,64]. On higher fat content mince compared to the lean (19.2% vs. 7.7%), enteric bacteria have also shown to be less inhibited by activities of LAB that can produce various antimicrobials and reduce the pH of meat during storage [57]. Typically, enteric bacteria spoil meat when the pH is ≥5.8 and temperatures >4 °C [65,66,67], but it has been shown that on red meat with pH around 5.5–6.0, similar to that observed here (Figure 3), some enteric bacteria (e.g., *H. alvei*, *Y. enterocolitica*, *Klebsiella pneumonia*, *Serratia* spp.) were causing premature spoilage at temperatures ranging between −1.5 and 7 °C [14,28,68]. These findings suggest that on high fat content mince, presumptive enteric bacteria had more favourable growth conditions with relatively lower lactic acid levels and higher pH compared to the control (Figure 2 and Figure 3). Additionally, lower glucose levels on high fat content mince (Figure 2) could have led enteric bacteria to earlier production of offensive odours through sulphurous compounds (e.g., acetoin, 1-octen-3-ol, diacetyl) and biogenic amines (e.g., putrescine, cadaverine, and tyramine) [69,70]. The increased availability of triglycerides on high fat content mince could have also promoted higher maximum populations of enteric bacterial strains (e.g., *Serratia* spp., *Yersinia* spp.) able to break down lipids with extracellular lipases and use glycerol as an energy source [40,71,72]. The role of extracellular lipases in increasing the maximum population and/or leading to earlier spoilage needs further research.

The varying fat content in mince could have also affected the successions of bacterial strains across time [73,74] and bacterial interactions within the community [60,75]. In the current study, the relationships between LAB and enteric bacteria could have led to higher presumptive enteric bacterial maximum population and/or faster growth rate of LAB on high fat content mince resulting in shorter shelf-life compared to the control. However, there are limited studies investigating these relationships between LAB and enteric bacteria on meat with varying fat content and how it influences the shelf-life determination. As the species of various bacteria were not identified here, further research is needed to determine what LAB and enteric species (and their possible interactions) could have been responsible for spoilage of red meat with higher fat content.

### 3.4. Limitations

One of the limitations of this study was that the lamb fat was autoclaved (121 °C for 25 min) to reduce the variations in the initial bacterial numbers of each treatment group as this was observed in the beef trial (Table 3). Specifically, the microbial numbers in beef mince appeared to increase with the 20% and 50% (*w*/*w*) treatments. The shelf-life of lamb mince may have been influenced by the sterilisation of lamb fat. Sterilisation can reduce the water content of the fat and could have caused oxidation and/or hydrolysis of fatty acid bonds. For example, heating at high temperatures (≥100 °C) can lead to the production of various lipid oxidation products (e.g., malondialdehyde, α-, β-unsaturated aldehydes, and volatile oxidation products) [76,77]. However, compared to oils, animal fats with high saturation levels have shown much lower concentrations of the oxidation products at high temperatures [76,77]. This suggests that the effects of sterilisation of lamb fat at 121 °C on shelf-life determination combined with immediate vacuum packing, may have been minimal. Moreover, the independent beef and lamb trials showed analogous results for shelf-life, glucose and lactic acid content, pH, and microbial growth kinetics. Specifically, lamb fat and beef fat had similar glucose and lactic acid contents (Figure 2), pH (Figure 3), and maximum TVC and presumptive enteric bacteria populations (Table 3). Previous work with chicken meat has also shown that bacterial growth rates do not change between cooked and raw meat [78]. Unlike raw meat, fat does not contain readily available carbohydrates (e.g., glucose in Figure 2) or proteins [39] that bacteria could degrade and cause off-odours [1,21,22]. Therefore, the authors believe that the sterilisation step did not impede comparisons between lamb treatments, although future studies with unsterilised fat are needed to confirm these findings.

## 4. Conclusions

This study provides scientific insights for the industry on how the fat content affects meat pH and its subsequent shelf-life by using red meat mince as model system for red meat primals. The results demonstrated that addition of fat (20% and 50% *w*/*w*) negatively impacted the shelf-life of VP beef and lamb mince at 2 °C when compared to their respective control (~5%). Although the apparent similarity in the shelf-lives of 20% and 50% mince for either beef or lamb could not be characterised, the shorter shelf-life of high fat content mince was accompanied by a decrease in glucose and lactic acid levels, combined with an increase in pH levels. These changes led to increased growth rates of LAB and a rise in the maximum populations of presumptive enteric bacteria, all of which ultimately act together to reduce the shelf-life of high fat content mince. However, future work using meat with different concentrations of fat is needed to specifically characterise the effects of fat on the shelf-life of VP red meat products. This study also did not differentiate LAB and presumptive enteric species and, therefore, further research is needed to determine how the specific species (and their possible interactions) affect spoilage of red meat with varying fat content.

## Figures and Tables

**Table 1:** Quantitation MRM and electrospray ionisation parameters of each analyte.

Analyte	Precursor [M-H]^−^ (M/Z) ^1^	Product [M-H]^−^ (M/Z) ^1^	Cone Voltage (V)	Collision Energy (V)	Limit of Detection in Meat (g/kg)
Lactic acid ^2^	89.1	43.0	24	12	0.00083
Glucose ^2^	179.1	45.0	16	16	0.00045
^3^H_2_-Lactic acid ^3^	92.1	45.0	24	12	-
^13^C_6_-Glucose ^3^	185.1	61.0	16	16	-

^1^ deprotonated ion measured by mass-to-charge ratio; ^2^ analyte measured in meat; ^3^ isotopically labelled standards, where limit of detection was not applicable (-).

**Table 2:** Average fat content (±standard deviation; wet *w*/*w*) of beef and lamb mince controls and treatments.

	Beef	Lamb
Lean mince (control)	4.31 ± 0.93%	5.35 ± 1.23%
20% mince	22.33 ± 7.02%	18.58 ± 4.78%
50% mince	44.09 ± 8.38%	43.82 ± 6.24%

**Table 3:** Summary of the bacterial growth kinetics of control (~5%), 20%, and 50% fat content (*w*/*w*) vacuum-packed beef and lamb mince. Initial and maximum bacterial populations are shown as the mean ± standard deviation of the count data (Supplementary Figure S2). Growth rates were calculated from regression lines fitted to respective growth curves (R^2^ ≥ 0.95) for each beef and lamb treatment.

		Total Viable Count			Lactic Acid Bacteria			Presumptive Enteric Bacteria ^1^	
		Initial Population (Log_10_ CFU/g)	Growth Rate (Log_10_ CFU/g/Day)	Maximum Population (Log_10_ CFU/g)	Initial Population (Log_10_ CFU/g)	Growth Rate (Log_10_ CFU/g/Day)	Maximum Population (Log_10_ CFU/g)	Initial Population (Log_10_ CFU/g)	Maximum Population (Log_10_ CFU/g)
Beef	Control (~5%)	4.59 ± 0.14 ^a^	0.43	8.22 ± 0.08 ^a^	3.69 ± 0.17 ^a^	0.27	7.70 ± 0.21 ^ac^	3.15 ± 0.32 ^ac^	5.06 ± 0.29 ^a^
	20%	4.89 ± 0.16 ^ab^	0.39	8.12 ± 0.15 ^ab^	4.11 ± 0.08 ^b^	0.34	7.59 ± 0.10 ^a^	3.39 ± 0.15 ^abc^	5.34 ± 0.26 ^ab^
	50%	5.00 ± 0.20 ^b^	0.34	8.00 ± 0.12 ^bc^	4.67 ± 0.27 ^c^	0.40	7.62 ± 0.24 ^a^	3.46 ± 0.06 ^abc^	5.54 ± 0.42 ^bf^
	Beef fat	5.29 ± 0.02 ^b^	ND	7.82 ± 0.16 ^c^	5.11 ± 0.03 ^d^	ND	6.88 ± 0.05 ^b^	3.86 ± 0.40 ^b^	6.36 ± 0.24 ^c^
Lamb	Control (~5%)	5.66 ± 0.15 ^c^	0.25	7.07 ± 0.20 ^d^	4.02 ± 0.03 ^ab^	0.31	6.98 ± 0.11 ^b^	3.15 ± 0.17 ^ac^	3.64 ± 0.18 ^d^
	20%	5.77 ± 0.02 ^c^	0.13	7.16 ± 0.12 ^d^	4.07 ± 0.06 ^b^	0.32	7.06 ± 0.18 ^b^	3.07 ± 0.21 ^c^	3.83 ± 0.26 ^d^
	50%	5.22 ± 0.07 ^b^	0.21	7.20 ± 0.17 ^d^	3.76 ± 0.11 ^ab^	0.35	7.07 ± 0.15 ^b^	3.38 ± 0.07 ^abc^	4.47 ± 0.27 ^e^
	Lamb fat	4.24 ± 0.06 ^e^	ND	7.93 ± 0.09 ^bc^	4.58 ± 0.03 ^c^	ND	8.02 ± 0.09 ^c^	3.65 ± 0.26 ^ab^	6.01 ± 0.11 ^cf^

^1^—presumptive enteric bacteria (*Enterobacteriaceae* family and its closely related organisms) colonies irrespective of their lactose metabolism; growth rates were not calculated due to very slow or no observed growth (Supplementary Figure S2); Different letters (^a–f^) in a column indicate statistically significant differences (*p* < 0.05) based on ANOVA pairwise comparisons; ND—not determined.

## Data Availability

The original contributions presented in the study are included in the article and Supplementary Materials, further inquiries can be directed to the corresponding author.

## Supplementary Materials

The following supporting information can be downloaded at: https://www.mdpi.com/article/10.3390/foods13223669/s1, Figure S1: Boxplots of odour scores for control (~5%), 20%, and 50% (*w*/*w*) fat content beef (on right) and lamb (on left) mince stored at 2 °C.; Figure S2: Total viable counts (TVC), lactic acid bacteria (LAB), and presumptive enteric bacteria (entero) counts on vacuum-packed beef (A, C, E) and lamb (B, D, F) mince with varying fat content stored at 2 °C.; Table S1: ANOVA-style summary of the six models of lactic acid and glucose content (g/kg), and fat content (%) for beef and lamb; Table S2: Contrasts corresponding to the GLS summarised in Supplementary Table S1 (without the interaction terms included) across all days.; Table S3: Contrasts between control (≤5%), 20%, and 50% beef mince pH from a series of linear mixed models (LMM) across storage time.; Table S4: Contrasts between control (≤5%), 20%, and 50% lamb mince pH from a series of linear mixed models (LMM) across storage time.
