# Supplementary material for: The Effects of Fat Content on the Shelf-Life of Vacuum-Packed Red Meat
Source: Foods. 2024 Nov 18;13(22):3669. doi: 10.3390/foods13223669 (PMC11594075; doi:10.3390/foods13223669)
Supplement: Supplementary file 1 [file foods-13-03669-s001.zip › suppl-figures-fatcontent.pdf]

Title: The effects of fat content on the shelf-life of vacuum-packed red meat
Supplementary figures:

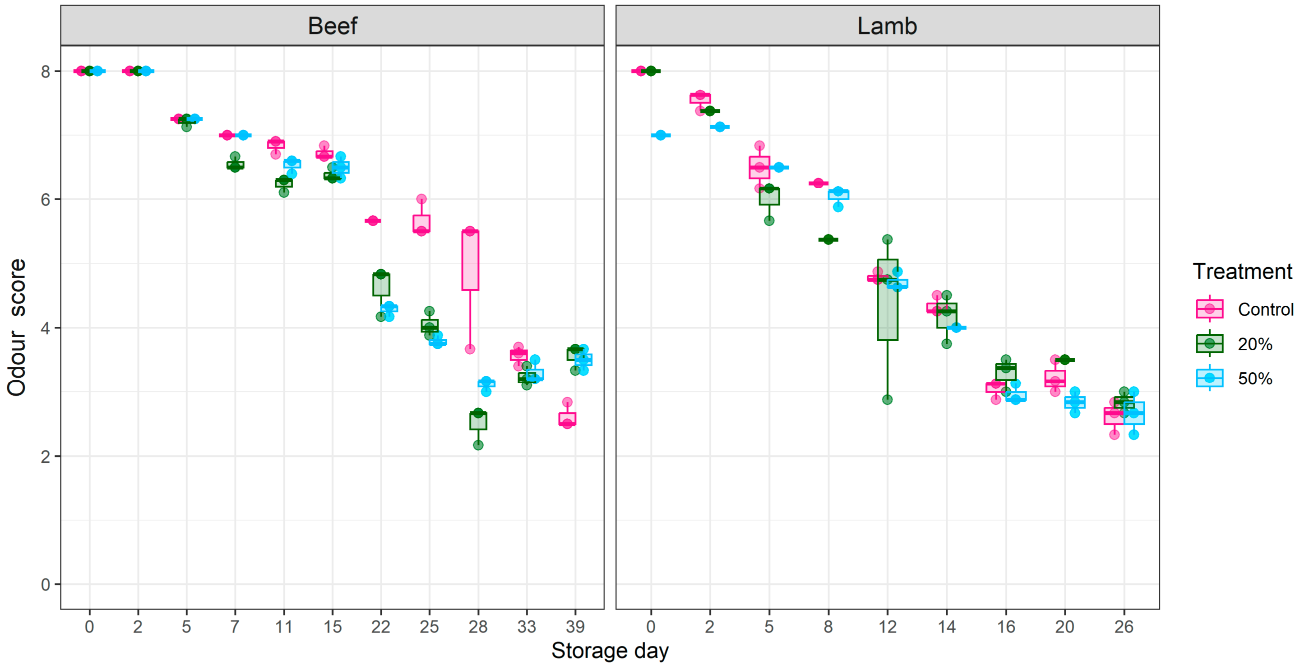

Figure S1. Boxplots of odour scores for control (~5 %), 20 %, and 50 % (w/w) fat content beef (on right) and lamb (on left) mince stored at 2°C. At each storage day three replicates per treatment were assessed, each data point is the average score given by the 3-6 semi-trained panellists. Odour score of 8 indicated no odour/normal meat odour and score of 0 the extreme off-odour. Samples with scores of ≤4 were deemed commercially unacceptable.

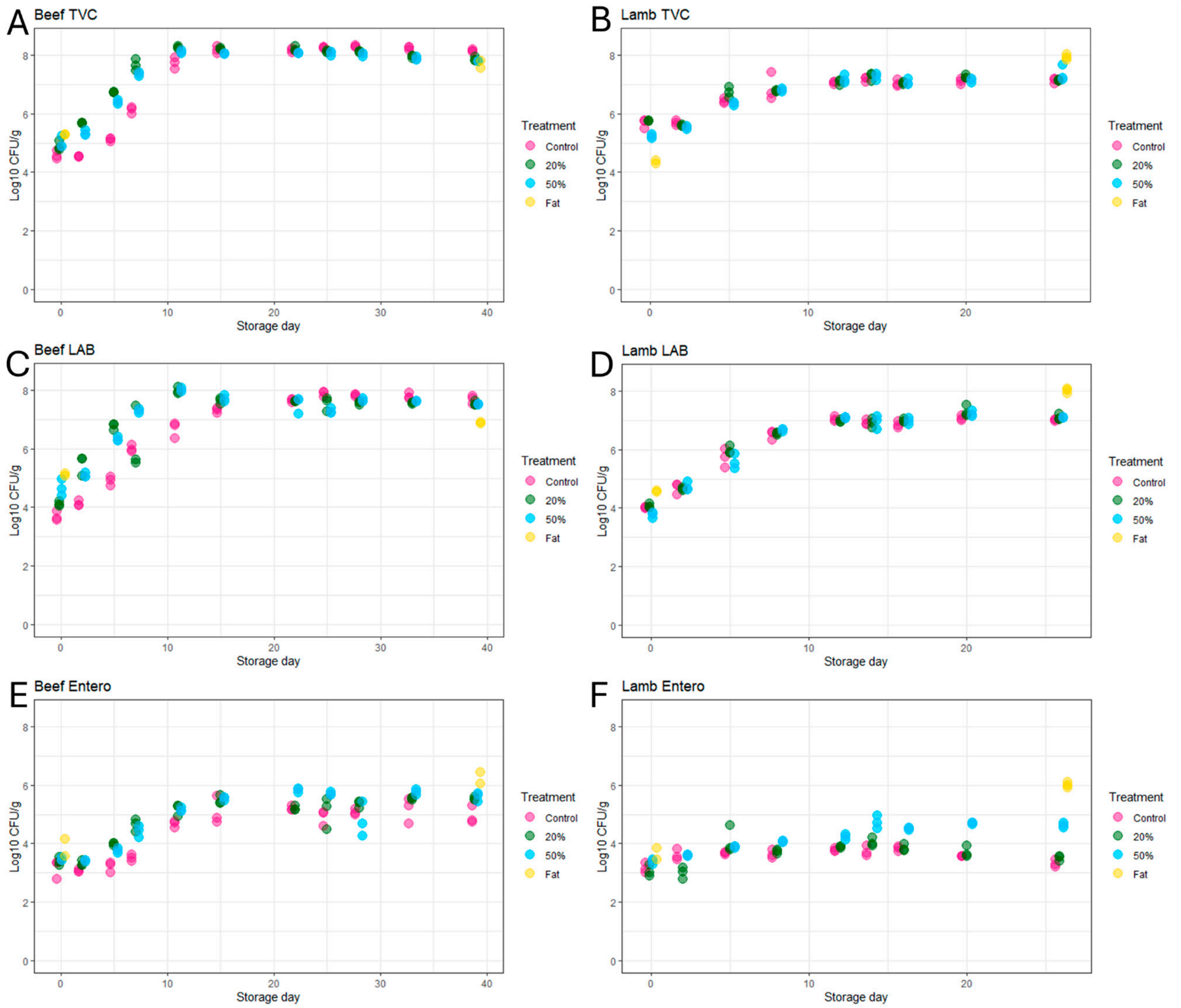

Figure S2. Total viable counts (TVC), lactic acid bacteria (LAB), and presumptive enteric bacteria (entero) counts on vacuum-packed beef (A, C, E) and lamb (B, D, F) mince with varying fat content stored at 2°C. Microbial load on fat samples was only measured at the start and end of the trials.
