# Supplementary material for: The Effects of Fat Content on the Shelf-Life of Vacuum-Packed Red Meat
Source: Foods. 2024 Nov 18;13(22):3669. doi: 10.3390/foods13223669 (PMC11594075; doi:10.3390/foods13223669)
Supplement: Supplementary file 1 [file foods-13-03669-s001.zip › supplementary-statstables-fatcontent.pdf]

Title: The effects of fat content on the shelf-life of vacuum-packed red meat

Table S1. ANOVA-style summary of the six models of lactic acid and glucose content (g/kg), and fat content (%) for beef and lamb. The base data for the lactic acid and glucose content regressions is shown in Figure 1 of the main text. Each model had the following x-variables: the storage day of sampling (categorical), the treatment (categorical, control (~5 %), 20 %, and 50 %) and an interaction term between these two variables. For each model, the variance of residual errors was estimated separately for each treatment. Each row of the table represents the results of an asymptotic Wald test, in which the null hypothesis is that the coefficients for the x-variable in that row are jointly zero (the Df column is the degrees of freedom applicable to the x-variable, the F-statistic and p-value correspond to the null hypothesis). The column of 'n' is the sample size for the model.

**Meat**	**y-variable**	**x-variable**	**Df**	**F- statistics**	**p-value**	**n**
**Beef**	Lactic acid	Day	2	6.079	0.010	27
	Lactic acid	Treatment	2	29.832	<0.001	27
	Lactic acid	Day:Treatment	4	0.177	0.947	27
	Glucose	Treatment	2	65.030	<0.001	9
	Fat	Day	2	0.439	0.651	27
	Fat	Treatment	2	76.954	<0.001	27
	Fat	Day:Treatment	4	0.470	0.757	27
**Lamb**	Lactic acid	Day	2	6.158	0.009	27
	Lactic acid	Treatment	2	70.355	<0.001	27
	Lactic acid	Day:Treatment	4	1.565	0.226	27
	Glucose	Day	1	581.472	<0.001	18
	Glucose	Treatment	2	52.264	<0.001	18
	Glucose	Day:Treatment	2	0.478	0.631	18
	Fat	Day	2	0.618	0.550	27
	Fat	Treatment	2	140.316	<0.001	27
	Fat	Day:Treatment	4	0.359	0.835	27

Table S2. Contrasts corresponding to the GLS summarised in Supplementary Table S1 (without the interaction terms included). The base data for the regressions is shown in Figure 1. The estimate column gives the mean difference between the two treatments in the contrast column (across all days). The test statistics and p-values correspond to asymptotic Wald tests for the null hypothesis that true difference in means between the pair of treatments is zero. The p-values have been adjusted via Bonferroni adjustments to reflect

the three multiple comparisons in each model. The last two columns are the lower and upper bounds of 95% confidence intervals for the estimated average mean difference.

Meat	Measure	Contrast (comparison group)	Estimated average difference	Test statistics	p-value	CI95% lower	CI95% upper
**Beef**	Lactic acid	20% - control	-1.090	-3.410	0.002	-1.717	-0.464
	Lactic acid	50% - control	-2.215	-8.313	<0.001	-2.738	-1.693
	Lactic acid	50% - 20%	-1.125	-3.032	0.007	-1.853	-0.398
	Glucose	20% - control	-0.041	-0.916	1.000	-0.129	0.047
	Glucose	50% - control	-0.162	-11.115	<0.001	-0.191	-0.134
	Glucose	50% - 20%	-0.121	-2.831	0.014	-0.205	-0.037
**Lamb**	Lactic acid	20% - control	-0.930	-3.621	0.001	-1.433	-0.426
	Lactic acid	50% - control	-2.656	-11.081	<0.001	-3.125	-2.186
	Lactic acid	50% - 20%	-1.726	-5.312	<0.001	-2.363	-1.089
	Glucose	20% - control	-0.097	-7.525	<0.001	-0.122	-0.072
	Glucose	50% - control	-0.224	-9.326	<0.001	-0.271	-0.177
	Glucose	50% - 20%	-0.127	-5.402	<0.001	-0.173	-0.081

Table S3. Contrasts between control (≤5%), 20%, and 50% beef mince pH from a series of linear mixed models (LMM) across storage time. For each day, a separate LMM is fitted. Each row in the table is defined by the day and contrast columns. The estimate column is based on the average differences in pH between the respective treatments. The p-values are from asymptotic Wald tests based on each model, for which the null hypothesis is that the difference between the respective two treatments is truly zero. The last two columns give associated 95% confidence intervals for the pH difference between treatments.

Storage day	Contrast (comparison group)	Estimated average difference	p-value	CI95% lower	CI95% upper
**0**	**20% - control**	0.009	0.700	-0.037	0.056
**2**	**20% - control**	-0.009	0.313	-0.027	0.009
**5**	**20% - control**	-0.050	<0.001	-0.072	-0.028
**7**	**20% - control**	-0.012	0.352	-0.036	0.013
**11**	**20% - control**	-0.132	<0.001	-0.176	-0.087
**15**	**20% - control**	0.020	0.131	-0.006	0.046
**22**	**20% - control**	0.120	<0.001	0.066	0.174
**25**	**20% - control**	0.030	0.268	-0.023	0.083
**28**	**20% - control**	0.140	0.001	0.056	0.224
**33**	**20% - control**	0.090	<0.001	0.049	0.131
**39**	**20% - control**	0.138	<0.001	0.081	0.194

**0**	**50% - control**	0.088	<0.001	0.042	0.135
**2**	**50% - control**	-0.010	0.271	-0.028	0.008
**5**	**50% - control**	-0.015	0.175	-0.037	0.007
**7**	**50% - control**	-0.008	0.550	-0.032	0.017
**11**	**50% - control**	-0.116	<0.001	-0.160	-0.071
**15**	**50% - control**	0.050	<0.001	0.024	0.076
**22**	**50% - control**	0.092	0.001	0.038	0.146
**25**	**50% - control**	0.027	0.325	-0.026	0.080
**28**	**50% - control**	0.078	0.072	-0.007	0.162
**33**	**50% - control**	0.122	<0.001	0.081	0.162
**39**	**50% - control**	0.069	0.016	0.013	0.125
**0**	**50% - 20%**	0.079	0.001	0.033	0.126
**2**	**50% - 20%**	-0.001	0.927	-0.019	0.017
**5**	**50% - 20%**	0.035	0.002	0.013	0.057
**7**	**50% - 20%**	0.004	0.740	-0.020	0.029
**11**	**50% - 20%**	0.016	0.485	-0.029	0.060
**15**	**50% - 20%**	0.030	0.024	0.004	0.056
**22**	**50% - 20%**	-0.028	0.304	-0.082	0.026
**25**	**50% - 20%**	-0.003	0.902	-0.056	0.050
**28**	**50% - 20%**	-0.063	0.147	-0.147	0.022
**33**	**50% - 20%**	0.032	0.128	-0.009	0.072
**39**	**50% - 20%**	-0.068	0.017	-0.124	-0.012

Table S4. Contrasts between control (≤5%), 20%, and 50% lamb mince pH from a series of linear mixed models (LMM) across storage time. For each day, a separate LMM is fitted. Each row in the table is defined by the day and contrast columns. The estimate column is based on the average differences in pH between the respective treatments. The p-values are for asymptotic Wald tests based on each model, for which the null hypothesis is that the difference between the respective two treatments is truly zero. The last two columns give associated 95% confidence intervals for the pH difference between treatments.

Storage day	Contrast (comparison group)	Estimated average difference	p-value	CI95% lower	CI95% upper
**0**	**20% - control**	0.048	0.054	-0.001	0.097
**2**	**20% - control**	0.017	0.215	-0.010	0.043
**5**	**20% - control**	0.039	0.001	0.016	0.062
**8**	**20% - control**	0.044	0.083	-0.006	0.094
**12**	**20% - control**	0.083	<0.001	0.053	0.114
**14**	**20% - control**	0.041	0.013	0.009	0.073
**16**	**20% - control**	0.029	0.328	-0.029	0.088

**20**	**20% - control**	0.054	0.004	0.017	0.091
**26**	**20% - control**	0.033	0.230	-0.021	0.086
**0**	**50% - control**	0.041	0.103	-0.008	0.090
**2**	**50% - control**	0.031	0.022	0.004	0.057
**5**	**50% - control**	0.068	<0.001	0.044	0.091
**8**	**50% - control**	0.108	<0.001	0.058	0.157
**12**	**50% - control**	0.159	<0.001	0.129	0.189
**14**	**50% - control**	0.119	<0.001	0.087	0.151
**16**	**50% - control**	0.125	<0.001	0.067	0.183
**20**	**50% - control**	0.135	<0.001	0.098	0.172
**26**	**50% - control**	0.176	<0.001	0.123	0.229
**0**	**50% - 20%**	-0.008	0.765	-0.057	0.042
**2**	**50% - 20%**	0.014	0.292	-0.012	0.041
**5**	**50% - 20%**	0.028	0.016	0.005	0.051
**8**	**50% - 20%**	0.063	0.013	0.013	0.113
**12**	**50% - 20%**	0.076	<0.001	0.046	0.106
**14**	**50% - 20%**	0.078	<0.001	0.046	0.111
**16**	**50% - 20%**	0.096	0.001	0.037	0.154
**20**	**50% - 20%**	0.081	<0.001	0.044	0.118
**26**	**50% - 20%**	0.143	<0.001	0.090	0.196
